# Supplementary material for: Genetic diversity and antifungal susceptibility among ecological niche populations of Aspergillus flavus in Yaoundé, Cameroon
Source: PLoS One. 2026 Sep 17;21(9):e0355362. doi: 10.1371/journal.pone.0355362 (PMC13585263; doi:10.1371/journal.pone.0355362)
Supplement: Supplementary file 1 [file pone.0355362.s002.docx]

**S1 Table**

| **Strain Number** | **Source** | ***AFLA1*** | ***AFLA3*** | ***AFLA7*** | ***AFPM3*** | ***AFPM4*** | ***AFPM7*** | **Antifungal MIC (VOR, ITR, TEB, DIF)** |
| --- | --- | --- | --- | --- | --- | --- | --- | --- |
| 196 | Groundnut | 6 | 7 | 10 | 14 | 12 | 26 | 0.5, 0.5, >4, 0.5 |
| 197 | Groundnut | 6 | 7 | 10 | 14 | 12 | 26 | 1, 0.5, 1, 1 |
| 208 | Groundnut | 6 | 7 | 10 | 18 | 12 | 0 | 0.5, 0.25, 0.5, 2 |
| 209 | Groundnut | 6 | 7 | 10 | 18 | 12 | 0 | 0.5, 0.5, 0.5, 2 |
| 210 | Groundnut | 3 | 7 | 4 | 10 | 8 | 0 | 1, 0.5, 2, 4 |
| 211 | Groundnut | 6 | 7 | 8 | 18 | 12 | 0 | 0.5, 1, 1, 2 |
| 232 | Groundnut | 6 | 7 | 10 | 14 | 12 | 26 | 0.5, 0.5, 2, 2 |
| 233 | Groundnut | 3 | 7 | 10 | 14 | 12 | 26 | 0.5, 0.5, 2, 2 |
| 236 | Groundnut | 6 | 7 | 10 | 14 | 12 | 26 | 0.5, 1, 1, 2 |
| 238 | Groundnut | 6 | 7 | 10 | 170* | 12 | 25 | 0.5, 0.5, 2, 2 |
| 239 | Groundnut | 6 | 7 | 10 | 14 | 12 | 27 | 1, 1, 1, 1 |
| 241 | Groundnut | 6 | 7 | 10 | 18 | 12 | 0 | 0.5, 1, 2, 1 |
| 242 | Groundnut | 3 | 7 | 10 | 18 | 12 | 0 | 0.5, 1, 1, 2 |
| 243 | Groundnut | 6 | 7 | 10 | 13 | 14 | 13 | 1, 0.5, 2, 2 |
| 257 | Groundnut | 6 | 7 | 10 | 18 | 12 | 0 | 1, 1, 1, 2 |
| 258 | Groundnut | 3 | 7 | 8 | 18 | 12 | 0 | 1, 1, 2, 2 |
| 259 | Groundnut | 25 | 7 | -8 | 18 | 12 | 0 | 1, 1, 2, 4 |
| 260 | Groundnut | 3 | 7 | 10 | 18 | 12 | 0 | 1, 1, 2, 4 |
| 261 | Groundnut | 2 | 7 | 8 | 18 | 12 | 0 | 0.5, 1, 1, 1 |
| 262 | Groundnut | 225* | 7 | 9 | 18 | 12 | 0 | 0.5, 1, 2, 4 |
| 263 | Groundnut | 6 | 7 | 8 | 18 | 12 | 0 | 0.5, 1, 2, 4 |
| 194 | Corn | 6 | 7 | 10 | 18 | 15 | 11 | 1, 1, 2, 2 |
| 195 | Corn | 6 | 7 | 8 | 13 | 8 | 10 | 1, 1, 4, 2 |
| 198 | Corn | 6 | 7 | 8 | 13 | 14 | 13 | 0.5, 1, 2, 2 |
| 199 | Corn | 6 | 7 | 10 | 13 | 14 | 13 | 0.5, 1, 4, 2 |
| 200 | Corn | 0 | 0 | 0 | 18 | 156* | 213* | 1, 0.5, 2, 1 |
| 201 | Corn | 6 | 7 | 10 | 18 | 12 | 0 | 0.5, 1, 2, 4 |
| 202 | Corn | 6 | 7 | 10 | 18 | 12 | 0 | 0.5, 2, 4, 1 |
| 203 | Corn | 9 | 7 | 9 | 17 | 15 | 10 | 0.25, 0.5, 1, 1 |
| 204 | Corn | 225* | 7 | 8 | 17 | 12 | 11 | 0.5, 0.5, 1, 2 |
| 205 | Corn | 225* | 7 | 0 | 18 | 19 | 12 | 0.5, >2, >4, 2 |
| 206 | Corn | 223* | 7 | 4 | 10 | 12 | 27 | 0.5, 0.5, 1, 1 |
| 207 | Corn | 6 | 7 | 10 | 18 | 12 | 0 | 0.5, 0.5, 0.5, 1 |
| 234 | Corn | 3 | 7 | 10 | 18 | 12 | 0 | 0.5, 1, 2, 2 |
| 235 | Corn | 6 | 7 | 10 | 18 | 12 | 0 | 0.5, 1, 0.5, 2 |
| 237 | Corn | 0 | 7 | 9 | 18 | 156* | 213* | 0.5, 1, 1, 2 |
| 244 | Corn | 3 | 7 | 10 | 18 | 12 | 0 | 1, 1, 2, 2 |
| 256 | Corn | 6 | 7 | 9 | 18 | 12 | 0 | 1, 0.5, 2, 1 |
| 264 | Corn | 6 | 7 | 8 | 18 | 8 | 10 | 0.5, 0.5, 1, 1 |
| 265 | Corn | 225* | 7 | 8 | 18 | 12 | 11 | 1, 1, 4, 4 |
| 266 | Corn | 6 | 7 | 4 | 18 | 12 | 0 | 1, 0.5, 4, 4 |
| 267 | Corn | 6 | 7 | 6 | 18 | 12 | 0 | 1, 1, 4, 4 |
| 268 | Corn | 6 | 7 | 10 | 18 | 12 | 0 | 1, 1, 4, 4 |
| 269 | Corn | 6 | 7 | 8 | 18 | 12 | 0 | 1, 0.5, 4, 4 |
| 91 | Air | 11 | 8 | 7 | 18 | 12 | 0 | 0.5, 2, 1, 1 |
| 93 | Air | 6 | 4 | 10 | 18 | 11 | 11 | 0.25, 2, 1, 1 |
| 99 | Air | 18 | 7 | 4 | 18 | 11 | 0 | 0.25, 0.5, 0.5, 0.5 |
| 104 | Air | 32 | 4 | 6 | 170* | 9 | 9 | 0.25, 0.5, 1, 1 |
| 115 | Air | 8 | 12 | 6 | 168* | 17 | 20 | 0.12, 0.5, 0.5, 0.5 |
| 123 | Air | 6 | 7 | 6 | 18 | 12 | 0 | 0.5, 0.5, 2, 1 |
| 151 | Air | 6 | 4 | 0 | 18 | 11 | 213* | 0.5, 1, 4, 1 |
| 152 | Air | 17 | 12 | 6 | 11 | 19 | 16 | 1, 1, 0.12, 1 |
| 14 | Human | 10 | 18 | 0 | 14 | 6 | 19 | 0.12, 1, 0.5, 0.25 |
| 97 | Human | 6 | 18 | 8 | 1 | 11 | 12 | 0.5, 1, 0.5, 0.12 |
| 13 | Soil | 30 | 18 | 6 | 1 | 8 | 23 | 0.5, 1, 1, 1 |
| 16 | Soil | 3 | 18 | 239* | 0 | 12 | 11 | 0.5, 0.5, 1, 1 |

*Used fragment size (bp) instead of repeat number due to potential deletion/shifts.

Note: Orange = resistant to either agricultural fungicide, Blue = resistant to itraconazole, VOR = Voriconazole, ITR = Itraconazole, TEB = Tebuconazole, DIF = Difenoconazole.
