# Supplementary material for: Genetic diversity and antifungal susceptibility among ecological niche populations of Aspergillus flavus in Yaoundé, Cameroon
Source: PLoS One. 2026 Sep 17;21(9):e0355362. doi: 10.1371/journal.pone.0355362 (PMC13585263; doi:10.1371/journal.pone.0355362)
Supplement: Supplementary file 2 [file pone.0355362.s003.docx]

**S2 Table**

| **Markers and Primer Sequence (5’ to 3’)** | **Repeat Unit** | **Repeat Number^1^** | **Fragment**  **Size (bp)^1^** |
| --- | --- | --- | --- |
| ***AFLA1*** | AC | 23 | 275 |
| CGTTGGCATGTTATCGTCAC |  |  |  |
| CTACTGAATGGCGGGACCTA |  |  |  |
| ***AFLA3*** | TAGG | 12 | 211 |
| CTGAAAGGGTAAGGGGAAGG |  |  |  |
| CACGCGAACTTATGGGACTT |  |  |  |
| ***AFLA7*** | TAG | 15 | 292 |
| GCGGACACTGGATGAATAGC |  |  |  |
| AACAAATCGGTGGTTGCTTC |  |  |  |
| ***AFPM3*** | (TG) (AT) 6  AAGGGCG (GA) | 16 | 204 |
| CCTTTCGCACTCCGAGAC |  |  |  |
| CACCACCAGTGATGAGGG |  |  |  |
| ***AFPM4*** | TG | 13 | 193 |
| AGCGATACAGTTTTAACACC |  |  |  |
| TCTTGCTATACATATCTTCACC |  |  |  |
| ***AFPM7*** | AC | 12 | 238 |
| TTGAGGCTGCTGTGGAACGC |  |  |  |
| CAAATACCAATTACGTCCAACAAGGG |  |  |  |

^1^Fragment size and repeat number were calculated using the NCBI *A. flavus* reference genome (RefSeq assembly GCF_009017415.1) or the NCBI sequence for locus AY510455 (GenBank). The repeat region was identified between the given primers to calculate repeat number.
