# Supplementary material for: Genetic diversity and antifungal susceptibility among ecological niche populations of Aspergillus flavus in Yaoundé, Cameroon
Source: PLoS One. 2026 Sep 17;21(9):e0355362. doi: 10.1371/journal.pone.0355362 (PMC13585263; doi:10.1371/journal.pone.0355362)
Supplement: Supplementary file 3 [file pone.0355362.s004.docx]

**S3 Table**

| **Markers** | **Multiplex PCR Fluorophore** | **Tail Sequence (5’ to 3’)** |
| --- | --- | --- |
| *AFLA1* | FAM, HEX | GCCTCCCTCGCGCCA |
| *AFLA3* | HEX | GCCTTGCCAGCCCGC |
| *AFLA7* | ATTO550, HEX | CAGGACCAGGCTACCGTG |
| *AFPM3* | FAM | GCCTCCCTCGCGCCA |
| *AFPM4* | HEX | GCCTTGCCAGCCCGC |
| *AFPM7* | ATTO550 | CAGGACCAGGCTACCGTG |
