# Supplementary material for: Genetic diversity and antifungal susceptibility among ecological niche populations of Aspergillus flavus in Yaoundé, Cameroon
Source: PLoS One. 2026 Sep 17;21(9):e0355362. doi: 10.1371/journal.pone.0355362 (PMC13585263; doi:10.1371/journal.pone.0355362)
Supplement: Supplementary file 4 [file pone.0355362.s005.docx]

**S4 Table**

| ***AMOVA and Recombination Test*** | Between ecological niche variation (%) | Within ecological niche variation (%) | Standardized Index of Association | Phylogenetic Compatibility (%) |
| --- | --- | --- | --- | --- |
|  | 17.54% | 82.46% | 0.223* | 0%* |

*p < 0.01
