# Supplementary material for: Genetic diversity and antifungal susceptibility among ecological niche populations of Aspergillus flavus in Yaoundé, Cameroon
Source: PLoS One. 2026 Sep 17;21(9):e0355362. doi: 10.1371/journal.pone.0355362 (PMC13585263; doi:10.1371/journal.pone.0355362)
Supplement: Supplementary file 5 [file pone.0355362.s006.docx]

**S5 Table**

| **Markers** | **Fragment Size Range (bp)** | **Repeat No. Range** |  | **No. of Unique Alleles** | | |  |  |
| --- | --- | --- | --- | --- | --- | --- | --- | --- |
|  |  |  | **Corn** | **Groundnut** | **Air** | **Soil** | **Human** | **Total** |
| ***AFLA1*** | 234 - 294 | 2 - 32 | 3 | 4 | 6 | 2 | 2 | 10 |
| ***AFLA3*** | 181 - 234 | 4 - 18 | 1 | 1 | 4 | 1 | 1 | 5 |
| ***AFLA7*** | 224 - 277 | -8 - 10 | 5 | 5 | 4 | 2 | 2 | 8 |
| ***AFPM3*** | 174 - 208 | 1 - 18 | 4 | 4 | 2 | 1 | 2 | 7 |
| ***AFPM4*** | 156 - 204 | -5 - 19 | 6 | 3 | 6 | 2 | 2 | 8 |
| ***AFPM7*** | 212 - 269 | -1 - 27 | 6 | 6 | 6 | 2 | 2 | 11 |
| **MIC**  **Range**  **(µg/mL)** | **Clinical** | **Voriconazole** | 0.25 - 1 | 0.5 - 1 | 0.12 - 1 | 0.5 | 0.12 - 0.5 | - |
|  |  | **Itraconazole** | 0.5 - > 2 | 0.25 - 1 | 0.5 - 2 | 0.5 - 1 | 1 |  |
|  | **Agricultural** | **Tebuconazole** | 0.5 - > 4 | 0.5 - > 4 | 0.12 - 4 | 1 | 0.5 |  |
|  |  | **Difenoconazole** | 1 - 4 | 0.5 - 4 | 0.5 - 1 | 1 | 0.12 – 0.25 |  |
|  | **Total No. of Isolates** | | **23** | **21** | **8** | **2** | **2** | **56** |
