# Supplementary material for: Genetic diversity and antifungal susceptibility among ecological niche populations of Aspergillus flavus in Yaoundé, Cameroon
Source: PLoS One. 2026 Sep 17;21(9):e0355362. doi: 10.1371/journal.pone.0355362 (PMC13585263; doi:10.1371/journal.pone.0355362)
Supplement: Supplementary file 6 [file pone.0355362.s007.docx]

## **S1 File**

## **Calculation of Repeat Number from Fragment Size**

Fragment size was identified for each marker on OSIRIS. Differences between the fragment size found in the isolate of study and the reference sequence were calculated. Using the size difference, the change in repeat number was found, which was used to determine the true repeat number in the isolate of study for each marker.

**M1-FAM (strain 196)**

Fragment Size on OSIRIS = 241

M1-FAM corresponds to marker *AFLA1*, which has a fragment size of 275 (Table. 2)

Fragment Size Difference = 275-241 = 34

*AFLA1* contains a dinucleotide repeat, thus 34 less base pairs in the isolate of study corresponds to 17 fewer repeats (34/2=17) (Table. 2)

No. of Repeats in Reference = 23 (Table. 2)

No. of Repeats in Strain 196 = 23-17 = 6

Thus, isolate 196 contains 6 repeats at the locus *AFLA1*
