# Supplementary material for: Genetic diversity and antifungal susceptibility among ecological niche populations of Aspergillus flavus in Yaoundé, Cameroon
Source: PLoS One. 2026 Sep 17;21(9):e0355362. doi: 10.1371/journal.pone.0355362 (PMC13585263; doi:10.1371/journal.pone.0355362)
Supplement: Supplementary file 7 [file pone.0355362.s009.docx]

August 27, 2026

Dear Editor and Reviewers,

Thank you for your time and expertise in helping us improve the study. We have now revised the manuscript addressing all comments raised by reviewers and the editorial office. The updated files have been uploaded to the journal website. Below are our responses to the specific comments.

**Journal Requirements:**

When submitting your revision, we need you to address these additional requirements.

1. Please ensure that your manuscript meets PLOS ONE's style requirements, including those for file naming. The PLOS ONE style templates can be found at

[https://journals.plos.org/plosone/s/file?id=wjVg/PLOSOne_formatting_sample_main_body.pdf](https://track.editorialmanager.com/CL0/https:%2F%2Fjournals.plos.org%2Fplosone%2Fs%2Ffile%3Fid=wjVg%2FPLOSOne_formatting_sample_main_body.pdf/1/010f01a0225cef46-7425c861-fce9-45dd-8a51-6b1c52c7fe98-000000/DmK5CzeqFkLOaUAIkewZl9Eowa9nLxI18Y2GGghwc5s=258) and

[https://journals.plos.org/plosone/s/file?id=ba62/PLOSOne_formatting_sample_title_authors_affiliations.pdf](https://track.editorialmanager.com/CL0/https:%2F%2Fjournals.plos.org%2Fplosone%2Fs%2Ffile%3Fid=ba62%2FPLOSOne_formatting_sample_title_authors_affiliations.pdf/1/010f01a0225cef46-7425c861-fce9-45dd-8a51-6b1c52c7fe98-000000/pOVcFGz5rgl7JNi19y0QBrumPL7lY5QNrA-rsr9WfFU=258)

**Response: We have changed the formatting to that suggested in the above linked document.**

2. Please note that PLOS One has specific guidelines on code sharing for submissions in which author-generated code underpins the findings in the manuscript. In these cases, we expect all author-generated code to be made available without restrictions upon publication of the work. Please review our guidelines at https://journals.plos.org/plosone/s/materials-and-software-sharing#loc-sharing-code and ensure that your code is shared in a way that follows best practice and facilitates reproducibility and reuse.

**Response: No applicable.**

3. In your Methods section, please provide additional information regarding the permits you obtained for the work. Please ensure you have included the full name of the authority that approved the field site access and, if no permits were required, a brief statement explaining why.

**Response: Done. We have added the permit details. The permits were already uploaded to the journal website during the initial submission.**

4. Please include a complete copy of PLOS’s questionnaire on inclusivity in global research in your revised manuscript. Our policy for research in this area aims to improve transparency in the reporting of research performed outside of the researchers’ own country or community. The policy applies to researchers who have travelled to a different country to conduct research, research with Indigenous populations or their lands, and research on cultural artefacts. The questionnaire can also be requested at the journal’s discretion for any other submissions, even if these conditions are not met.  Please find more information on the policy and a link to download a blank copy of the questionnaire here: https://journals.plos.org/plosone/s/best-practices-in-research-reporting. Please upload a completed version of your questionnaire as Supporting Information when you resubmit your manuscript.

**Response: Done. A completed questionnaire file has been uploaded.**

5. We note that the grant information you provided in the ‘Funding Information’ and ‘Financial Disclosure’ sections do not match.

When you resubmit, please ensure that you provide the correct grant numbers for the awards you received for your study in the ‘Funding Information’ section.

**Response: Done. We have ensured the information is consistent across the fields.**

6. Thank you for stating the following financial disclosure:

“This research was supported by grants from McMaster Global Science Initiative (2021-03) and the Aspergillosis Trust (Charity Number 1194699).”

Please state what role the funders took in the study.  If the funders had no role, please state: "The funders had no role in study design, data collection and analysis, decision to publish, or preparation of the manuscript."

If this statement is not correct you must amend it as needed.

Please include this amended Role of Funder statement in your cover letter; we will change the online submission form on your behalf.

**Response: Done. We have modified the cover letter to include that "The funders had no role in study design, data collection and analysis, decision to publish, or preparation of the manuscript."**

7. Please note that funding information should not appear in any section or other areas of your manuscript. We will only publish funding information present in the Funding Statement section of the online submission form. Please remove any funding-related text from the manuscript.

**Response: Done. The Funding Information section has been removed from the manuscript.**

8. Please include your full ethics statement in the ‘Methods’ section of your manuscript file. In your statement, please include the full name of the IRB or ethics committee who approved or waived your study, as well as whether or not you obtained informed written or verbal consent. If consent was waived for your study, please include this information in your statement as well.

**Response: The required statements have been provided.**

9. We note you have included a table to which you do not refer in the text of your manuscript. Please ensure that you refer to Table A5 in your text; if accepted, production will need this reference to link the reader to the Table.

**Response: Done. This has been added to the manuscript text.**

10. If the reviewer comments include a recommendation to cite specific previously published works, please review and evaluate these publications to determine whether they are relevant and should be cited. There is no requirement to cite these works unless the editor has indicated otherwise. 
**Response: Not Applicable.**

11. Please review your reference list to ensure that it is complete and correct. If you have cited papers that have been retracted, please include the rationale for doing so in the manuscript text, or remove these references and replace them with relevant current references. Any changes to the reference list should be mentioned in the rebuttal letter that accompanies your revised manuscript. If you need to cite a retracted article, indicate the article’s retracted status in the References list and also include a citation and full reference for the retraction notice.

**Response: The referees didn’t suggest any specific reference. However, five new references have been added, all of which were related to addressing reviewers’ comments. As far as we know, none of the papers we cited have been retracted.**

[Note: HTML markup is below. Please do not edit.]

Reviewers' comments:

Reviewer's Responses to Questions

**Comments to the Author**

1. Is the manuscript technically sound, and do the data support the conclusions?

The manuscript must describe a technically sound piece of scientific research with data that supports the conclusions. Experiments must have been conducted rigorously, with appropriate controls, replication, and sample sizes. The conclusions must be drawn appropriately based on the data presented.

Reviewer #1: Yes

Reviewer #2: Yes

2. Has the statistical analysis been performed appropriately and rigorously?

Reviewer #1: Yes

Reviewer #2: Yes

3. Have the authors made all data underlying the findings in their manuscript fully available?

The [PLOS Data policy](http://track.editorialmanager.com/CL0/http:%2F%2Fwww.plosone.org%2Fstatic%2Fpolicies.action%23sharing/1/010f01a0225cef46-7425c861-fce9-45dd-8a51-6b1c52c7fe98-000000/da8MJA2_5dVC2zVYqHxcS2V_OL40ScR81QLz-XPtDM0=258) requires authors to make all data underlying the findings described in their manuscript fully available without restriction, with rare exception (please refer to the Data Availability Statement in the manuscript PDF file). The data should be provided as part of the manuscript or its supporting information, or deposited to a public repository. For example, in addition to summary statistics, the data points behind means, medians and variance measures should be available. If there are restrictions on publicly sharing data—e.g. participant privacy or use of data from a third party—those must be specified.

Reviewer #1: Yes

Reviewer #2: Yes

4. Is the manuscript presented in an intelligible fashion and written in standard English?

PLOS ONE does not copyedit accepted manuscripts, so the language in submitted articles must be clear, correct, and unambiguous. Any typographical or grammatical errors should be corrected at revision, so please note any specific errors here.

Reviewer #1: Yes

Reviewer #2: Yes

5. Review Comments to the Author

Please use the space provided to explain your answers to the questions above. You may also include additional comments for the author, including concerns about dual publication, research ethics, or publication ethics. (Please upload your review as an attachment if it exceeds 20,000 characters)

Reviewer #1: The studies investigated the prevalence of triazoles resistance in clinical and agricultural strains of Aspergillus flavus from Cameroon. The results showed the presence of resistance to 3 triazoles. Agricultural strains had the highest cross resistance to fungicides. The significant correlations in growth of some strains under both agricultural and clinic triazole is concerning.
The paper is well written, and the results would be of interest to the readership of PLOS ONE.
Minor corrections
The introduction should provide information on: (1) the crops sprayed with the triazole in Cameroon, and (2) the recommended rates for the different clinal and agricultural triazoles?
**Response: Thanks for your comments and suggestions. We have now listed a few major crops that are known to be sprayed on in Cameroon and the major issues with their applications of agrochemicals, including fungicides such as difenoconazole and tebuconazole in Cameroon (lines 113-122). Three new references (reference numbers 20, 21, and 22) are added to address your comments and to briefly describe the scale of the issue there.**

Lines 139-152: Where were the air samples collected from? The market, open fields, forest or houses?

**Response: Air samples were collected within hospital wards (line 145).**

Line 463: ‘Given…’ instead of ‘Giving…’

**Response: Done. This has been changed (line 439).**

Line 463-464: Discuss how sampling from Yaoundé market rather regional markets or farms may have impacted your results.

**Response: Thanks for your suggestion. We have now added the following to the discussion. “**We note that the crop samples were obtained in markets in the capital Yaoundé and not from farms where the crops were grown. This was mainly done for two reasons. The first was that the capital markets receive agricultural products from farms in diverse regions in Cameroon, thus the sampled crop products are more likely to represent the broader *A. flavus* contaminations in Cameroon than individual farms. The second reason was that these crop products were what people in Yaoundé were exposed, which could have direct health consequences for residents in the capital. The findings of high genetic diversity among strains from corn and groundnut samples individually are consistent with their diverse origins of *A. flavus*. Sampling local farms may reveal limited genetic variations among strains of *A. flavus* within each farm but potentially genetic differentiation among *A. flavus* populations from different farms and regions.” (lines 441-451).

Conclusion: While I see the need for more regulation on use of fungicides, I am concerned that more stringent controls on fungicides would lead to great losses to the farmers due to lack of equally effective alternatives. The authors should include examples of biocontrol products that can be used instead of the agricultural triazoles in the conclusion.

**Response: Thanks for bringing this up. We have now included an additional statement and two new references here to indicate a number of alternatives to conventional chemical fungicides, including biological control agents on crop fungal pathogens and contaminants (lines 418-425; new references 44, and 45).**

Reviewer #2: Aspergillus flavus is a ubiquitous filamentous mold and poses a significant threat to human health through both food contamination and human infection. Therefore, investigating antifungal resistance in this species is of great importance. This study is well conducted and clearly written. However, only one strain was found to be resistant to itraconazole. It is unclear whether this strain also exhibited cross-resistance to difenoconazole or tebuconazole. The authors should clarify this point, if such data are available.

**Response: Thanks for your comment. This point has been clarified. The itraconazole-resistant strain was also resistant to tebuconazole (MIC > 4µg/mL) but not to difenoconazole (MIC = 2µg/mL). (lines 247-249).**

6. PLOS authors have the option to publish the peer review history of their article ([what does this mean?](https://track.editorialmanager.com/CL0/https:%2F%2Fjournals.plos.org%2Fplosone%2Fs%2Feditorial-and-peer-review-process%23loc-peer-review-history/1/010f01a0225cef46-7425c861-fce9-45dd-8a51-6b1c52c7fe98-000000/tjAXchZdtdF-oRZd9CRWCaVBj93NaDKiq0mv24PFnsM=258)). If published, this will include your full peer review and any attached files.

If you choose “no”, your identity will remain anonymous but your review may still be made public.

**Do you want your identity to be public for this peer review?** For information about this choice, including consent withdrawal, please see our [Privacy Policy](https://track.editorialmanager.com/CL0/https:%2F%2Fwww.plos.org%2Fprivacy-policy/1/010f01a0225cef46-7425c861-fce9-45dd-8a51-6b1c52c7fe98-000000/td7OV9cHaicpA-volfxBAIISTl-7rYbBFDQ-MKLT9qI=258).

Reviewer #1: **Yes:** Emma W. Gachomo

Reviewer #2: No
