# Supplementary material for: Genetic diversity and antifungal susceptibility among ecological niche populations of Aspergillus flavus in Yaoundé, Cameroon
Source: PLoS One. 2026 Sep 17;21(9):e0355362. doi: 10.1371/journal.pone.0355362 (PMC13585263; doi:10.1371/journal.pone.0355362)
Supplement: Peer review history

# Peer review: Decision Letter 0

**DOI:** 10.1371/journal.pone.0355362.r001

**Authors:** Katherine Borkovich

20 Aug 2026

Dear Dr. Xu,

Thank you for submitting your manuscript to PLOS One. After careful consideration, we feel that it has merit but does not fully meet PLOS One’s publication criteria as it currently stands. Therefore, we invite you to submit a revised version of the manuscript that addresses the points raised during the review process.  You should incorporate the suggestions of the two expert reviewers while revising your manuscript.

Please submit your revised manuscript by Sep 19 2026 11:59PM. If you will need more time than this to complete your revisions, please reply to this message or contact the journal office at plosone@plos.org. When you're ready to submit your revision, log on to https://www.editorialmanager.com/pone/ and select the 'Submissions Needing Revision' folder to locate your manuscript file.

- A letter that responds to each point raised by the academic editor and reviewer(s). You should upload this letter as a separate file labeled 'Response to Reviewers'.
- A marked-up copy of your manuscript that highlights changes made to the original version. You should upload this as a separate file labeled 'Revised Manuscript with Track Changes'.
- An unmarked version of your revised paper without tracked changes. You should upload this as a separate file labeled 'Manuscript'.

If you would like to make changes to your financial disclosure, please include your updated statement in your cover letter. Guidelines for resubmitting your figure files are available below the reviewer comments at the end of this letter.

If applicable, we recommend that you deposit your laboratory protocols in protocols.io to enhance the reproducibility of your results. Protocols.io assigns your protocol its own identifier (DOI) so that it can be cited independently in the future. For instructions see: https://journals.plos.org/plosone/s/submission-guidelines#loc-laboratory-protocols. Additionally, PLOS One offers an option for publishing peer-reviewed Lab Protocol articles, which describe protocols hosted on protocols.io. Read more information on sharing protocols at https://plos.org/protocols?utm_medium=editorial-email&utm_source=authorletters&utm_campaign=protocols.

As the corresponding author, your ORCID iD is verified in the submission system and will appear in the published article. PLOS supports the use of ORCID, and we encourage all coauthors to register for an ORCID iD and use it as well. Please encourage your coauthors to verify their ORCID iD within the submission system before final acceptance, as unverified ORCID iDs will not appear in the published article. *Only*  the individual author can complete the verification step; PLOS staff *cannot*  verify ORCID iDs on behalf of authors.

We look forward to receiving your revised manuscript.

Kind regards,

Katherine A. Borkovich, Ph.D.

Academic Editor

PLOS One

**Journal Requirements:**

When submitting your revision, we need you to address these additional requirements.

1. Please ensure that your manuscript meets PLOS ONE's style requirements, including those for file naming. The PLOS ONE style templates can be found at

https://journals.plos.org/plosone/s/file?id=wjVg/PLOSOne_formatting_sample_main_body.pdf and

https://journals.plos.org/plosone/s/file?id=ba62/PLOSOne_formatting_sample_title_authors_affiliations.pdf

2. Please note that PLOS One has specific guidelines on code sharing for submissions in which author-generated code underpins the findings in the manuscript. In these cases, we expect all author-generated code to be made available without restrictions upon publication of the work. Please review our guidelines at https://journals.plos.org/plosone/s/materials-and-software-sharing#loc-sharing-code and ensure that your code is shared in a way that follows best practice and facilitates reproducibility and reuse.

3. In your Methods section, please provide additional information regarding the permits you obtained for the work. Please ensure you have included the full name of the authority that approved the field site access and, if no permits were required, a brief statement explaining why.

4. Please include a complete copy of PLOS’s questionnaire on inclusivity in global research in your revised manuscript. Our policy for research in this area aims to improve transparency in the reporting of research performed outside of the researchers’ own country or community. The policy applies to researchers who have travelled to a different country to conduct research, research with Indigenous populations or their lands, and research on cultural artefacts. The questionnaire can also be requested at the journal’s discretion for any other submissions, even if these conditions are not met.  Please find more information on the policy and a link to download a blank copy of the questionnaire here: https://journals.plos.org/plosone/s/best-practices-in-research-reporting. Please upload a completed version of your questionnaire as Supporting Information when you resubmit your manuscript.

5. We note that the grant information you provided in the ‘Funding Information’ and ‘Financial Disclosure’ sections do not match.

When you resubmit, please ensure that you provide the correct grant numbers for the awards you received for your study in the ‘Funding Information’ section.

6. Thank you for stating the following financial disclosure:

“This research was supported by grants from McMaster Global Science Initiative (2021-03) and the Aspergillosis Trust (Charity Number 1194699).”

Please state what role the funders took in the study.  If the funders had no role, please state: "The funders had no role in study design, data collection and analysis, decision to publish, or preparation of the manuscript."

If this statement is not correct you must amend it as needed.

Please include this amended Role of Funder statement in your cover letter; we will change the online submission form on your behalf.

7. Please note that funding information should not appear in any section or other areas of your manuscript. We will only publish funding information present in the Funding Statement section of the online submission form. Please remove any funding-related text from the manuscript.

8. Please include your full ethics statement in the ‘Methods’ section of your manuscript file. In your statement, please include the full name of the IRB or ethics committee who approved or waived your study, as well as whether or not you obtained informed written or verbal consent. If consent was waived for your study, please include this information in your statement as well.

9. We note you have included a table to which you do not refer in the text of your manuscript. Please ensure that you refer to Table A5 in your text; if accepted, production will need this reference to link the reader to the Table.

10. If the reviewer comments include a recommendation to cite specific previously published works, please review and evaluate these publications to determine whether they are relevant and should be cited. There is no requirement to cite these works unless the editor has indicated otherwise.

11. Please review your reference list to ensure that it is complete and correct. If you have cited papers that have been retracted, please include the rationale for doing so in the manuscript text, or remove these references and replace them with relevant current references. Any changes to the reference list should be mentioned in the rebuttal letter that accompanies your revised manuscript. If you need to cite a retracted article, indicate the article’s retracted status in the References list and also include a citation and full reference for the retraction notice.

[Note: HTML markup is below. Please do not edit.]

Reviewers' comments:

Reviewer's Responses to Questions

**Comments to the Author**

1. Is the manuscript technically sound, and do the data support the conclusions?

Reviewer #1: Yes

Reviewer #2: Yes

**********

2. Has the statistical analysis been performed appropriately and rigorously?

Reviewer #1: Yes

Reviewer #2: Yes

**********

3. Have the authors made all data underlying the findings in their manuscript fully available?

The PLOS Data policy

Reviewer #1: Yes

Reviewer #2: Yes

**********

4. Is the manuscript presented in an intelligible fashion and written in standard English?

Reviewer #1: Yes

Reviewer #2: Yes

**********

Reviewer #1: The studies investigated the prevalence of triazoles resistance in clinical and agricultural strains of Aspergillus flavus from Cameroon. The results showed the presence of resistance to 3 triazoles. Agricultural strains had the highest cross resistance to fungicides. The significant correlations in growth of some strains under both agricultural and clinic triazole is concerning.

The paper is well written, and the results would be of interest to the readership of PLOS ONE.

Minor corrections

The introduction should provide information on: (1) the crops sprayed with the triazole in Cameroon, and (2) the recommended rates for the different clinal and agricultural triazoles?

Lines 139-152: Where were the air samples collected from? The market, open fields, forest or houses?

Line 463: ‘Given…’ instead of ‘Giving…’

Line 463-464: Discuss how sampling from Yaoundé market rather regional markets or farms may have impacted your results.

Conclusion: While I see the need for more regulation on use of fungicides, I am concerned that more stringent controls on fungicides would lead to great losses to the farmers due to lack of equally effective alternatives. The authors should include examples of biocontrol products that can be used instead of the agricultural triazoles in the conclusion.

Reviewer #2: Aspergillus flavus is a ubiquitous filamentous mold and poses a significant threat to human health through both food contamination and human infection. Therefore, investigating antifungal resistance in this species is of great importance. This study is well conducted and clearly written. However, only one strain was found to be resistant to itraconazole. It is unclear whether this strain also exhibited cross-resistance to difenoconazole or tebuconazole. The authors should clarify this point, if such data are available.

**********

what does this mean?). If published, this will include your full peer review and any attached files.

If you choose “no”, your identity will remain anonymous but your review may still be made public.

**Do you want your identity to be public for this peer review?**  For information about this choice, including consent withdrawal, please see our Privacy Policy

Reviewer #1: **Yes:** Emma W. Gachomo

Reviewer #2: No

**********

[NOTE: If reviewer comments were submitted as an attachment file, they will be attached to this email and accessible via the submission site. Please log into your account, locate the manuscript record, and check for the action link "View Attachments". If this link does not appear, there are no attachment files.]

To ensure your figures meet our technical requirements, please review our figure guidelines: https://journals.plos.org/plosone/s/figures

You may also use PLOS’s free figure tool, NAAS, to help you prepare publication quality figures: https://journals.plos.org/plosone/s/figures#loc-tools-for-figure-preparation.

NAAS will assess whether your figures meet our technical requirements by comparing each figure against our figure specifications.

# Author comment: Author response to Decision Letter 1

**DOI:** 10.1371/journal.pone.0355362.r002

29 Aug 2026

Dear Reviewers,

Thank you for your time and expertise in helping us improve the study. We have now revised the manuscript addressing all comments raised by reviewers and the editorial office. The updated files have been uploaded to the journal website. The locations of the changes are listed as line numbers in the clean revised version. Below are our responses to the specific comments.

Reviewer #1: The studies investigated the prevalence of triazoles resistance in clinical and agricultural strains of Aspergillus flavus from Cameroon. The results showed the presence of resistance to 3 triazoles. Agricultural strains had the highest cross resistance to fungicides. The significant correlations in growth of some strains under both agricultural and clinic triazole is concerning.

The paper is well written, and the results would be of interest to the readership of PLOS ONE.

Minor corrections

The introduction should provide information on: (1) the crops sprayed with the triazole in Cameroon, and (2) the recommended rates for the different clinal and agricultural triazoles?

Response: Thanks for your comments and suggestions. We have now listed a few major crops that are known to be sprayed on in Cameroon and the major issues with their applications of agrochemicals, including fungicides such as difenoconazole and tebuconazole in Cameroon (lines 113-122). Three new references (reference numbers 20, 21, and 22) are added to address your comments and to briefly describe the scale of the issue there.

Lines 139-152: Where were the air samples collected from? The market, open fields, forest or houses?

Response: Air samples were collected within hospital wards (line 145).

Line 463: ‘Given…’ instead of ‘Giving…’

Response: Done. This has been changed (line 439).

Line 463-464: Discuss how sampling from Yaoundé market rather regional markets or farms may have impacted your results.

Response: Thanks for your suggestion. We have now added the following to the discussion. “We note that the crop samples were obtained in markets in the capital Yaoundé and not from farms where the crops were grown. This was mainly done for two reasons. The first was that the capital markets receive agricultural products from farms in diverse regions in Cameroon, thus the sampled crop products are more likely to represent the broader A. flavus contaminations in Cameroon than individual farms. The second reason was that these crop products were what people in Yaoundé were exposed, which could have direct health consequences for residents in the capital. The findings of high genetic diversity among strains from corn and groundnut samples individually are consistent with their diverse origins of A. flavus. Sampling local farms may reveal limited genetic variations among strains of A. flavus within each farm but potentially genetic differentiation among A. flavus populations from different farms and regions.” (lines 441-451).

Conclusion: While I see the need for more regulation on use of fungicides, I am concerned that more stringent controls on fungicides would lead to great losses to the farmers due to lack of equally effective alternatives. The authors should include examples of biocontrol products that can be used instead of the agricultural triazoles in the conclusion.

Response: Thanks for bringing this up. We have now included additional statements and two new references here to indicate a number of alternatives to conventional chemical fungicides, including biological control agents on crop fungal pathogens and contaminants (lines 418-425; new references 44, and 45).

Reviewer #2: Aspergillus flavus is a ubiquitous filamentous mold and poses a significant threat to human health through both food contamination and human infection. Therefore, investigating antifungal resistance in this species is of great importance. This study is well conducted and clearly written. However, only one strain was found to be resistant to itraconazole. It is unclear whether this strain also exhibited cross-resistance to difenoconazole or tebuconazole. The authors should clarify this point, if such data are available.

Response: Thanks for your comment. This point has been clarified. The itraconazole-resistant strain was also resistant to tebuconazole (MIC > 4µg/mL) but not to difenoconazole (MIC = 2µg/mL). (lines 247-249).

Attachment Submitted filename: PLOS One review and response letter.docx

# Editor report: Decision Letter 1

**DOI:** 10.1371/journal.pone.0355362.r003

**Authors:** Katherine Borkovich

2 Sep 2026

Genetic Diversity and Antifungal Susceptibility among Ecological Niche Populations of Aspergillus flavus in Yaoundé, Cameroon

PONE-D-26-36147R1

Dear Dr. Xu,

We’re pleased to inform you that your manuscript has been judged scientifically suitable for publication and will be formally accepted for publication once it meets all outstanding technical requirements.

Within one week, you’ll receive an e-mail detailing the required amendments. When these have been addressed, you’ll receive a formal acceptance letter and your manuscript will be scheduled for publication.

An invoice will be generated when your article is formally accepted. Please note, if your institution has a publishing partnership with PLOS and your article meets the relevant criteria, all or part of your publication costs will be covered. Please make sure your user information is up-to-date by logging into Editorial Manager at Editorial Manager® and clicking the ‘Update My Information' link at the top of the page. For questions related to billing, please contact billing support.

If your institution or institutions have a press office, please notify them about your upcoming paper to help maximize its impact. If they’ll be preparing press materials, please inform our press team as soon as possible -- no later than 48 hours after receiving the formal acceptance. Your manuscript will remain under strict press embargo until 2 pm Eastern Time on the date of publication. For more information, please contact onepress@plos.org.

Kind regards,

Katherine A. Borkovich, Ph.D.

Academic Editor

PLOS One

Additional Editor Comments (optional):

Reviewers' comments:

# Editor report: Acceptance letter

**DOI:** 10.1371/journal.pone.0355362.r004

**Authors:** Katherine Borkovich

PONE-D-26-36147R1

PLOS One

Dear Dr. Xu,

I'm pleased to inform you that your manuscript has been deemed suitable for publication in PLOS One. Congratulations! Your manuscript is now being handed over to our production team.

At this stage, our production department will prepare your paper for publication. This includes ensuring the following:

* All references, tables, and figures are properly cited

* All relevant supporting information is included in the manuscript submission,

* There are no issues that prevent the paper from being properly typeset

You will receive further instructions from the production team, including instructions on how to review your proof when it is ready. Please keep in mind that we are working through a large volume of accepted articles, so please give us a few days to review your paper and let you know the next and final steps.

Lastly, if your institution or institutions have a press office, please let them know about your upcoming paper now to help maximize its impact. If they'll be preparing press materials, please inform our press team within the next 48 hours. Your manuscript will remain under strict press embargo until 2 pm Eastern Time on the date of publication. For more information, please contact onepress@plos.org.

You will receive an invoice from PLOS for your publication fee after your manuscript has reached the completed accept phase. If you receive an email requesting payment before acceptance or for any other service, this may be a phishing scheme. Learn how to identify phishing emails and protect your accounts at https://explore.plos.org/phishing.

If we can help with anything else, please email us at customercare@plos.org.

Thank you for submitting your work to PLOS One and supporting open access.

Kind regards,

PLOS One Editorial Office Staff

on behalf of

Dr. Katherine A. Borkovich

Academic Editor

PLOS One
